# Retraction: The Ratio of Circulating Regulatory T Cells (Tregs)/Th17 Cells Is Associated with Acute Allograft Rejection in Liver Transplantation

**DOI:** 10.1371/journal.pone.0222348

**Published:** 2019-09-05

**Authors:** The PLOS ONE Editors

Concerns have been raised that the transplants performed in the local context of procedures reported in this article [1] may have involved organs/tissues procured from prisoners [2].

The authors reported that the transplants investigated in this work were obtained from deceased donors. However, further details as to the donor sources and methods of obtaining informed consent from donors were not reported in [1], and the authors did not reply to journal queries seeking to clarify these issues and the cause(s) of donor death. International ethics standards call for transparency in organ donor and transplantation programs and clear informed consent procedures including considerations to ensure that donors are not subject to coercion.

In addition, the authors did not provide documentation when requested by the journal to confirm that the study had institutional ethics approval; they did not report participant information in the Methods such as the location and dates of recruitment, dates of transplant procedures, and the inclusion/exclusion criteria applied; and they did not provide the primary data underlying this study’s results as needed to comply with the PLOS Data Availability Policy.

Owing to the lack of documentation to demonstrate this study had prospective ethical approval, insufficient reporting, unresolved concerns around the source of transplanted organs and whether they included organs from prisoners, and in compliance with international ethical standards for organ/tissue donation and transplantation, the *PLOS ONE* Editors retract this article.

MS agreed with the retraction. YW, MZ, ZWL, WGR, YCS, YLS, HBW, LJ, and FSW did not respond.
